# Prediction equation to estimate heart rate at individual ventilatory threshold in female and male obese adults

**DOI:** 10.1371/journal.pone.0197255

**Published:** 2018-05-11

**Authors:** Gian Pietro Emerenziani, Dafne Ferrari, Maria Grazia Vaccaro, Maria Chiara Gallotta, Silvia Migliaccio, Andrea Lenzi, Carlo Baldari, Laura Guidetti

**Affiliations:** 1 Department of Experimental and Clinical Medicine, University of Catanzaro "Magna Græcia", Catanzaro, Italy; 2 Department of Movement, Human and Health Sciences, Section of Health Sciences, University of Rome “Foro Italico”, Rome, Italy; 3 Institute of Neurology, Department of Medical Sciences, University of Catanzaro “Magna Græcia”, Catanzaro, Italy; 4 Department of Experimental Medicine, Section of Medical Pathophysiology, Food Science and Endocrinology, Sapienza University, Rome, Italy; Rutgers University Newark, UNITED STATES

## Abstract

**Objective:**

Prescribing individualized moderate exercise intensity is a useful method to reach positive effects on health status in obese adults. This study aimed to establish a practical reference equation to estimate the heart rate (HR) at individual ventilatory threshold (IVT) (HR_IVT_).

**Methods:**

One hundred sixty-one obese subjects were clinically evaluated and characterized by anthropometric and body composition. Participants performed the six-minute walking test (6-MWT) and the cardiopulmonary exercise test to assess IVT. Multiple regression analysis for HR_IVT_, including 6-MWT, anthropometric, and body composition parameters, as independent variables, was performed for both gender separately. A cross-validation study was also performed to determine the accuracy of the prediction equation.

**Results:**

Whereas HR_IVT_ was not significantly different between males (121.5±18.3 bpm) and females (117.6±17.1 bpm), it differently correlated with physical and performance parameters. Therefore, two sex-specific equations were developed including 6-MWT_HR_ and HR_rest_ (R^2^ = 0.69 and 0.65 and root mean square errors of 8.8 and 10.1 bpm for females and males, respectively).

**Conclusion:**

In conclusion, in female and male obese adults, the 6-MWT might be used to predict HR at IVT. These outcomes are useful to prescribe optimal physical activity intensity when gold standard methods (e.g. gas exchange analysis) are unavailable.

## Introduction

Obesity, one of the most relevant health problems of modern society [1], is a chronic condition characterized by an excess of adipose tissue and it is generally defined by body mass index (BMI) equal to or greater than 30 kg/m^2^ [1]. However, since BMI does not reflect the correct amount of body adipose tissue other parameters, such as body fat mass (%FM), are used in order to correctly define obesity [2].

Lave et al. [2] suggest, as a cut-off point for obesity diagnosis, values of %FM ranging from 23%-24% in men and 30%-35% in women. Therefore, the combined use of both BMI and %FM values might be a better approach to evaluate health risks related to obesity.

Interestingly, scientific evidence [3] suggests a correlation between obesity and functional limitation in both motor activity and sense of fatigue, which may contribute to physical inactivity and to a reduced exercise tolerance [4].

It is generally accepted that regular physical activity (PA) provides health benefits and it is proposed as a component of primary and secondary prevention for most of metabolic chronic diseases [5]. Furthermore, it has been demonstrated that subjects that increase their level of PA over time have decreased mortality rate as compared to those that were unfit [6]. Moreover, it has been shown that endurance and endurance strength training had positive effects on body composition, physical capacity, and renal function in women with abdominal obesity [7,8].

The role of exercise intensity on physical training adherence, and on achieving goals is supported by several large studies [9,10].

In this sense, it becomes particularly relevant to individualize the exercise intensity accordingly to the real subjects’ abilities, leading to increase their physical fitness and improve quality of life [11]. Thus, individual ventilatory threshold (IVT) has been considered as useful tool for prescribing correct exercise intensity in obese population [12].

Previous studies demonstrate that exercise intensity based upon IVT may lead to a reduction of body weight, resulting in an increase of physical performance in obese population [13–16].

Cardiopulmonary exercise test (CPET) has been used to assess IVT however, this practice requires qualified technical staff and sophisticated equipment. Therefore, only qualified and/or trained staff in a laboratory environment can assess IVT. Although the CPET remains the gold-standard method to assess subjects’ cardiorespiratory parameters, the high-intensity nature of exhaustive stress tests might be inappropriate for obese patients and for subjects with low fitness levels. Therefore, it would be very interesting to use a field test, which does not require expensive device, to evaluate functional status and to provide information regarding the optimal exercise intensity that obese adults should use to maximize the positive effects of PA. In particular, six-minute walking test (6-MWT) [17] has been used to estimate physical capacities in obese individuals [13,17]. In fact, the 6-MWT is a practical field test habitually used to assess walking capacity, and its results are highly correlated with cardiovascular tests [17]. However, additional data linking the 6-MWT performance to selected physiological measures of cardiopulmonary exercise test are needed to increase the potential use of this field test. To our knowledge, the possibility of a relationship between 6-MWT and the heart rate corresponding to IVT (HR_IVT_) has never been verified, and the prediction of HR_IVT_ by anthropometric and body composition parameters and 6-MWT has never been obtained. It is noteworthy to highlight the importance of the correlations of these tests in obese subjects, in order to lead exercise specialists to plan correctly the training program for this population in a widespread manner.

Therefore, the aim of this study was to establish a practical reference equation to facilitate the prediction of the HR_IVT_ in female and male obese adult subjects.

## Materials and methods

### Subjects

One hundred sixty-one (not practicing any physical activity) obese adults (116 females; 45 males) (age = 47.3±12.3 years, BMI = 38.2±6.3 kg/m^2^) were recruited in this study from patients admitted to the Day Hospital of the Department of Experimental Medicine, Section of Medical Pathophysiology, Endocrinology & Nutrition, Policlinico Umberto I, “Sapienza” University of Rome. All participants underwent clinical examination to exclude any contraindications to PA. Inclusion criteria consisted of: adult age (>18 years), BMI ≥ 30 kg/m^2^ and fat mass ≥35% in females and ≥25% in males [1,2]. The exclusion criteria were: neuropathy, autonomic dysfunction, cardiovascular diseases (myocardial infarction during the previous six months, myocardial ischaemia or ventricular tachycardia, obstructive valvular heart disease), uncontrolled hypertension (blood pressure values exceeding 140 mm Hg systolic or 90 mm Hg diastolic). Moreover, independent samples of sixty-six females (age = 46.0 ±11.3 years, BMI = 38.2±6.0 kg/m^2^; %FM = 45.5±4.3%, VO_2peak_ = 20.5±4.2 ml/kg/min) and twenty-eight males (age = 49.0±12.2 years, BMI = 38.2±4.3 kg/m^2^; %FM = 34.7±3.9%, VO_2peak_ = 2325±4.0 ml/kg/min) were selected for cross-validation analysis. These subjects were recruited using the same inclusion/exclusion criteria and from the same Day Hospital. Each participant provided a written informed consent before the participation in the study. This study was conducted according to the Declaration of Helsinki and was approved by the Sapienza /University of Rome Ethical Committee (approval number 70/11, 2011).

### Anthropometric and body composition measurements

Height (to nearest 0.1 cm) and weight (to nearest 0.02 cm) were measured using a stadiometer. Body mass index (BMI) was calculated dividing body weight in kilograms by height in meters squared (kg/m^2^). Body composition (fat and fat free mass) was evaluated by DEXA Discovery (Hologic, Bedford, United States). Variables analyzed were fat mass and fat free mass. Percent of body fat (%FM) and percent of fat free mass (%FFM) were calculated.

### Physiological and functional measurements

After a familiarization session, subjects performed a 6-MWT and a graded exercise test (GXT) on a treadmill. All tests were performed in the morning. Functional evaluation measurements were performed at the Department of Movement, Human and Health Sciences at the University of Rome “Foro Italico”. Each subject rested quietly for 5 min to properly assess resting heart rate (HR). The mean HR of the last 30sec was taken as the resting value (HR_rest_). Maximal HR (HR_max_) was calculated as previously described (HR_max_ = 208–0.7·age) [18]. Then, subjects performed 6-MWT and an incremental GXT on a treadmill in a randomized way. No subjects reported pain or dyspnea following the 6-MWT. Between the end of 6-MWT and the beginning of incremental test a minimum of 30 minutes rest were given to subjects to avoid the effects of fatigue and muscle soreness. During rest period subjects were asked to lie on a bed until HR returned to the resting value. The 6-MWT was carried out in a 50-meter corridor marked every 5 meters with tapes on the floor. Subjects were permitted to stop or rest during the test if severe health problems (such as strong muscle pain or cardiac impairments) has accursed. Standardized encouragements were provided at recommended intervals. Distance walked (6-MWT_dist_) and mean HR during 6-MWT (6-MWT_HR_) were measured by using a HR monitor (RS 400, Polar Electro™, Kempele, Finland); gait speed (6-MWT_speed_) was calculated [(distance/360)·3.6]. Our internal laboratory data showed that CV and ICC of 6-MWT were 1.4% and 0.99, respectively.

Peak oxygen consumption (VO_2peak_) was assessed in all participants by means of a continuous, incremental GXT on a treadmill (Woodway PRO, Woodway, Waukesha, WI, USA). Our internal laboratory data showed that CV and ICC of graded exercise test were 4.7% and 0.98, respectively. VO_2_, carbon dioxide production (VCO_2_), and ventilation (VE) were measured by an automatic gas analyzer (Quark RMR-CPET Cosmed™, Rome, Italy) [19], which was calibrated before each test in accordance with the manufacturer’s instructions.

The continuous graded incremental treadmill test was conducted as described in our previous published study [13]. In details, the incremental test started at 3 km/h. The speed was increased by 1 km/h every three minutes until 5 km/h was reached and then the slope was increased by 3% every three minutes until one of the following conditions was reached: a value of 10 on RPE-OMNI-Walk/Run Scale [20] or the subject’s HR reached a value of 90% of their predicted HR_max_. HR (beats/min) was continuously recorded before and throughout the trial using a HR monitor (RS 400, Polar Electro™, Kempele, Finland). Before the test, a standard definition of perceived exertion along with the OMNI-Walk/Run Scale was explained to the participant, while, during the test the participant was asked to report the rate of perceived exertion (RPE) on the OMNI Walk/Run scale every 2 minutes, according to the instructions described by Utter et al. [21].

During the test, the highest VO_2_ attained was chosen as the VO_2peak_. The individual ventilatory threshold (IVT) was determined offline by two independent peers for each subject by plotting the ventilatory equivalent of oxygen (VE/VO_2_) as a function of VO_2_ to identify mathematically and visually the point where this curve reached its lowest value during the exercise test [22]. The workload at which we observed the lowest value of the VE/VO_2_, in the individual plot, was the individual ventilatory threshold [12–14,22].

Our internal laboratory data showed that CV and ICC of IVT were 4.3% and 0.97, respectively. The exercise intensity corresponding to IVT was reached by all the subjects. At IVT and at maximal effort, HR and metabolic equivalent (MET) were determined. In addition, %HR_max_ [(HR at IVT)/(220-age)] was also calculated.

### Statistical analysis

It was calculated that a sample size of at least 42 subjects in each gender group would yield at least 80% power of detecting an intervention effect (ES = 0.25) that was statistically significant at the 0.05 α level. The Kolmogorov-Smirnov test was used to ensure normally distributed data. All data are presented as mean values ± standard deviation (SD). Differences between sexes were evaluated with an unpaired 𝑡-test. Due to significant sex-differences in several variables, the regression analysis was performed separately in females and males.

Correlation analysis was used to explore the relationships between HR_IVT_ and the measured variables. Stepwise regression analysis was performed to identify which combination of significantly related variables would best predict HR_IVT_ measured by CPET. The coefficient of determination (R^2^) and the SEE were estimated. The criterion for inclusion (addition and retention) of predictors was the highest R^2^ model and the lowest SEE. Statistical significance was assumed at the conventional level of p ≤ 0.05. In the current study, cross-validation of predicted equations was performed by using the root mean squared error (RMSE) methods [23] to an independent sample. We checked the non-differences between validation and cross-validation groups with unpaired 𝑡-tests and no significant differences were found for all the measured variables. RMSE is a measure of the performance of prediction equation when applied to an independent sample. It is calculated as the square root of the sum of squared differences between the observed and the predicted values divided by the number of subjects in the cross-validation sample. The smaller RMSE, the greater the accuracy of the equation and it should be less than 10% of the range of target property value [23].

All statistical analyses were performed with the SPSS statistical package (Version 24.0 for Windows; SPSS Inc., Chicago, IL, USA).

## Results

### Anthropometric and body composition variables

Anthropometric characteristics and body composition data of obese subjects are shown in Table 1. As expected, height, weight, FFM, and %FFM were significantly higher (P < 0.01) in male than in female subjects, while %FM was significantly lower in male than in female individuals.

**Table 1:** Subject physical characteristics. Table’s values are expressed as mean ± standard deviation (SD).

Parameters	Female (N = 116)	Male (N = 45)	P value
Age (years)	46.5 ± 12.4	49.5 ± 12.0	P = 0.39
Height (m)	1.60 ± 0.06	1.73 ± 0.07**	P<0.01
Weight (kg)	98.7 ± 18.8	113.8 ± 19.1**	P<0.01
BMI (kg/m)	38.3 ± 6.5	37.8 ± 5.5	P = 0.69
FFM (kg)	52.2 ± 8.1	72.1 ± 10.2**	P<0.01
%FM (%)	46.3 ± 4.7	35.8 ± 4.5**	P<0.01
%FFM (%)	53.7 ± 5.4	64.2 ± 7.1**	P<0.01

BMI = body mass index; FFM = fat-free mass expressed in kg; FM = fat mass.

** Statistically significant vs female (P < 0.01).

### 6-MWT and cardiopulmonary exercise tests

All participants completed both 6-MWT and CPET tests without premature conclusion or breaks, and no complications occurred during assessments. Results of these tests are depicted in Table 2. Briefly, total distance covered during the 6-MWT was significantly higher (P <0.01) in male than in female subjects but there was no significant difference in HR average during test. During 6-MWT no subjects has stopped for adverse health problems.

**Table 2:** Physiological parameters at rest, peak, individual ventilatory threshold (IVT) and six minute walking test (6-MWT) results.

Parameters	Female (N = 116)	Male (N = 45)	P value
*At rest*			
HR_rest_	74.3 ± 10.7	74.5 ± 11.5	P = 0.89
*At peak*			
VO_2peak_ (ml/kg/min)	20.4 ± 3.9	22.6 ± 4.2*	P = 0.02
VO_2peak_ (ml/min)	1987.7 ± 426.1	2544.0 ± 495.8**	P<0.01
MET_peak_	5.8 ± 1.1	6.5 ± 1.2*	P = 0.02
VE_peak_ (l/min)	59.1 ± 13.7	72.8 ± 19.0**	P<0.01
HR_peak_ (bpm)	145.8 ± 20.6	142.0 ± 20.9	P = 0.30
%HR_max_ (%)	82.9 ± 8.6	81.8 ± 10.3	P = 0.52
*At IVT*			
VO_2IVT_ (ml/kg/min)	14.4 ± 3.2	15.0 ± 2.6	P = 0.26
VO_2IVT_ (ml/min)	1416.6 ± 387.9	1671.8 ± 441.0**	P<0.01
MET_IVT_	4.1 ± 0.9	4.3 ± 0.7	P = 0.26
VE_IVT_ (l/min)	34.6 ± 9.5	39.5 ± 8.8	P = 0.08
HR_IVT_ (bpm)	121.5 ± 18.3	117.6 ± 17.1	P = 0.22
%HR_max_ (%)	69.1 ± 9.0	67.9 ± 9.9	P = 0.43
6-MWT			
Distance (m)	557.6 ± 68.5	606.7 ± 67.9**	P<0.01
Gait Speed (km/h)	1.55 ± 0.19	1.69 ± 0.19**	P<0.01
HR_mean_ (bpm)	130.3 ±19.7	130.6 ± 17.9	P = 0.02

VO_2_: oxygen uptake; MET: metabolic equivalent; VE: ventilation; HR: heart rate; %HR_max_: heart rate expressed as percentage of HR_max_ (208–0.7·age); HR_rest_: heart rate at rest.

* Statistically significant vs female (P < 0.05).

** Statistically significant vs female (P < 0.01).

Regarding the results of CPET males showed better performance than females, as depicted in Table 2. In details, a significant higher (P <0.01) VO_2,_ MET and VE at peak were observed in males than in females. At IVT males showed a significant higher VO_2_ expressed as ml/min than females.

### Stepwise and multiple regression analyses between HR at IVT and independent variables

**Fig 1:**
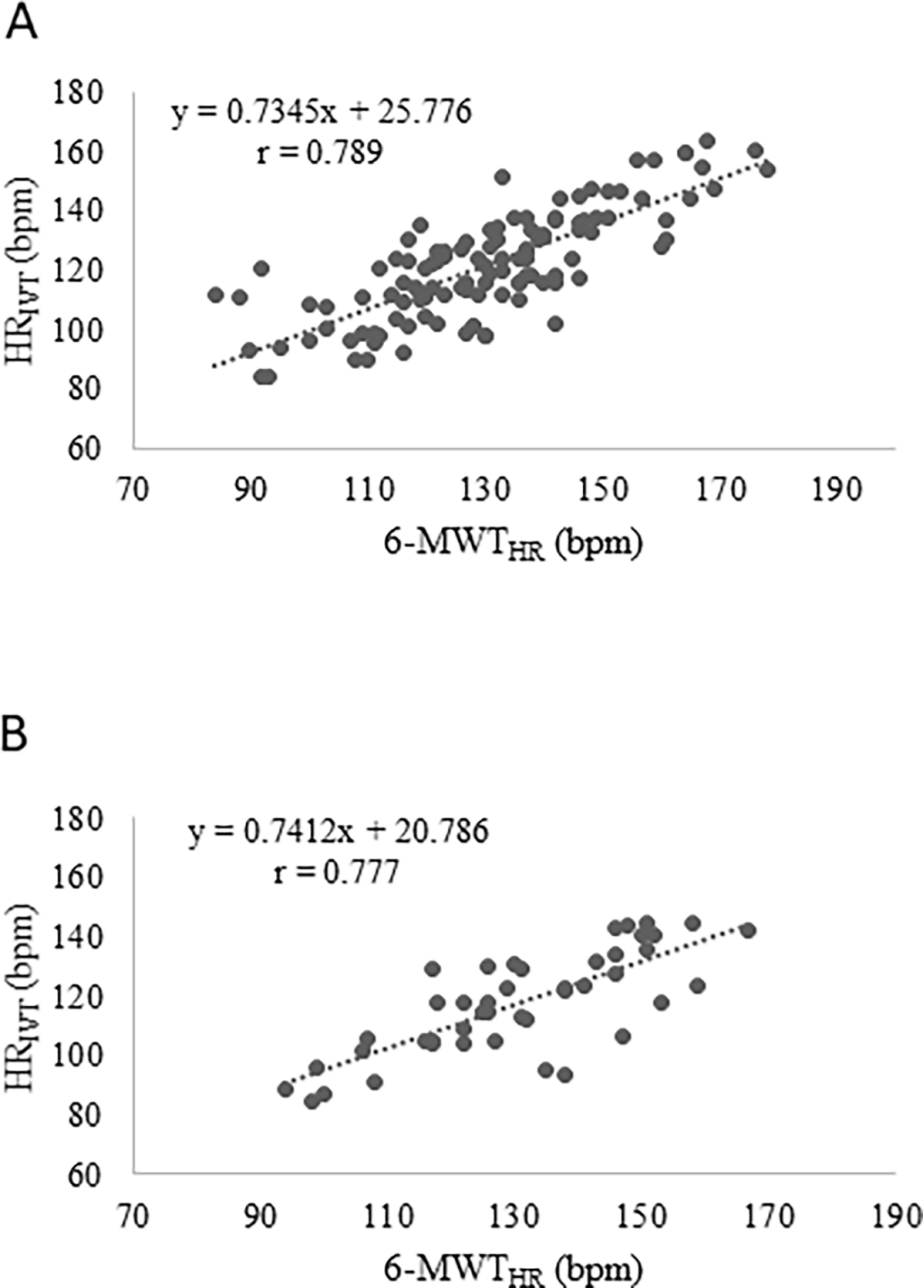
Scatterplot of HR at IVT (HR_IVT_) to mean HR during 6-MWT (6-MWT_HR_) for females (A) and males (B).

**Fig 2:**
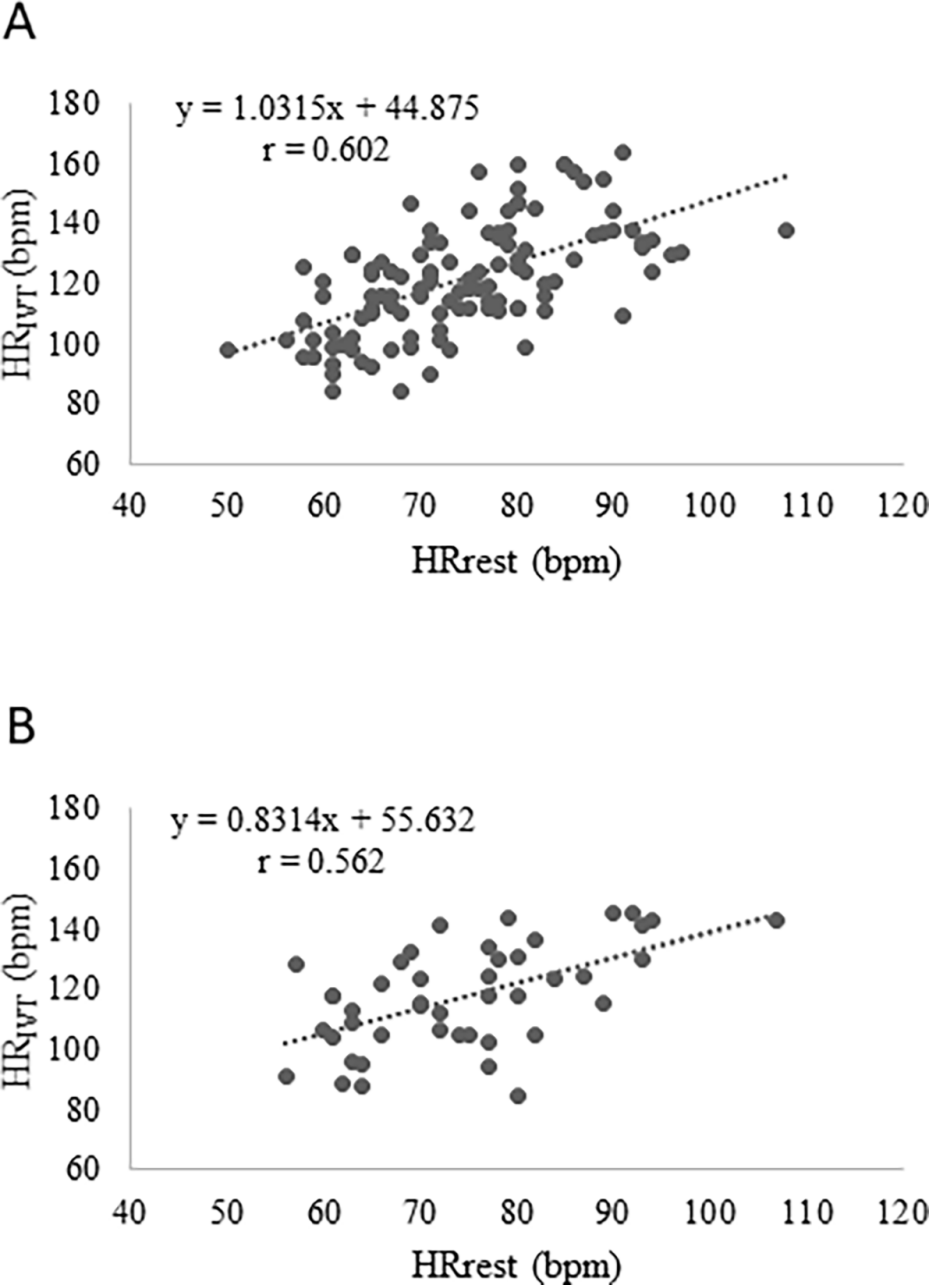
Scatterplot of HR at IVT (HR_IVT_) to resting HR (HR_rest_) for females (A) and males (B).

Height, weight, BMI, fat free mass, HR_max_, HR_rest_, and 6-MWT_HR_, significantly correlated with HR_IVT_ in females (Table 3) while BMI, %FM, HR_rest_, and 6-MWT_HR_ in males (Table 3). HR_IVT_ showed significant negative correlation with age (r = -0.504) in females and with %FFM (r = -0.323) in males. 6-MWT_HR_ (Fig 1) and HR_rest_ (Fig 2) displayed the highest correlation with HR_IVT_ in both gender. The results of the stepwise multiple regressions showed that 6-MWT_HR_, and HR_rest_ data can give the best predictive model in both females (r = 0.83, R^2^ = 0.69) and males (r = 0.80, R^2^ = 0.65) as shown in Table 4. We assessed multicollinearity between HR_IVT_ and 6-MWT_HR_ by examining tolerance and the Variance Inflation Factor (VIF). In our regression models, tolerance was 0.760 in males and 0.773 in females and VIF was equal to 1.316 in males and 1.294 in females.

**Table 3:** Variables significantly correlated to HR_IVT_.

	R	P
*Female*		
Age (years)	-0.504	≤ 0.01
Height (m)	0.190	0.041
Weight (kg)	0.280	≤ 0.01
BMI (kg/m^2^)	0.216	≤ 0.01
FFM (kg)	0.258	≤ 0.01
HR_max_ (bpm)	0.504	≤ 0.01
HR_rest_ (bpm)	0.602	≤ 0.01
6-MWT_HR_ (bpm)	0.789	≤ 0.01
*Male*		
HR_rest_ (bpm)	0.562	≤ 0.01
BMI (kg/m^2^)	0.314	0.036
%FM (%)	0.373	0.012
%FFM (%)	- 0.323	0.030
6-MWT_HR_ (bpm)	0.777	≤ 0.01

BMI = body mass index; FM = fat mass; FFM = fat free mass; HR = heart rate; 6MWT six minute walking test.

**Table 4:** Multiple regression equations predicting HR at IVT in both sexes.

	Coefficient	SE	R2	SEE	P
Female (n = 181)					
Constant	5.459	7.672			
HRrest (bpm)	0.501	0.102			
6-MWTHR (bpm)	0.605	0.056			
Total model			0.688	10.34	<0.001
Male (n = 55)					
Constant	9.037	12.636			
HRrest (bpm)	0.354	0.155			
6-MWTHR (bpm)	0.629	0.100			
Total model			0.647	10.38	<0.001

HRrest = heart rate at rest; HRmean 6-MWTHR = average heart rate during six minute walking test.

From the result of multiple regression analysis the prediction equations to estimate HR_IVT_ are:

For Females: HR_IVT_ = 5.459 + (0.501 HR_rest_) + (0.605 6-MWT_HR_)

For Males: HR_IVT_ = 9.037 + (0.354 HR_rest_) + (0.629 6-MWT_HR_)

For cross-validation analysis, prediction equations were used on sixty-six females and twenty-eight males. Subjects’ HR_IVT_ were 118.3 ± 14.3 bpm and 114.4 ± 12.0 bpm in females and males respectively. Moreover, predicted mean HR_IVT_ was 122.4 ± 13.8 bpm in females and 113.2 ± 10.8 in males. The HR_IVT_ values for the RMSE were 8.8 bpm in females and 10.1 bpm for males. Therefore, RMSE was 7% and 9% of the range of target property value in females and males respectively.

## Discussion

In this study, we evaluate the relationship between HR_IVT_, body composition and 6-MWT for the purpose of establishing a practical reference equation facilitating the prediction of HR_IVT_ in obese adult subjects of both gender. Our current study findings demonstrate for the first time that, from a multivariable model, 6-MWT_HR_ and the HR_rest_ were predictive of heart rate at individual ventilatory threshold in obese adults in both female and male individuals.

Subjects involved in the study were affected by obesity and they had not practiced any organized PA prior to the enrolment in this study. VO_2peak_ results (20.4 and 22.6 ml/kg/min in females and males respectively) indicate that subjects were unfit, a common situation in obese population, as previously described [14,24,25]. Significant differences in body composition were found between females and males. In details, as expected, at the same BMI, men had lower %FM and higher %FFM, weight and height as compared to women. These differences may justify the significant higher VO_2_, MET, and VE at peak in male than female individuals.

In this study, during GXT, subjects did not reach their theoretical maximal HR (HR_max_ = 208–0.7·age) [18] but they stopped around 83% and 82% in females and males respectively. Moreover, subjects reached an RPE value equal to 10 before that their HR was closed to HR_max_. This result might be justified by the impairment due to body size and low level of fitness and probably because the participants, having poorly previously practiced PA, were not aware of their actual physical capacities. These results are indeed in agreement with previous studies [12,25] which demonstrated that when exercise intensity is prescribed only accordingly to parameters based upon the estimated HR_max_ (such as %HR_max_), without adjustment for individual’s exercise capacity, exercise intensity might not be individually appropriate for obese subjects.

As expected, a difference between males and females was found in the 6-MWT test, as also previously reported in the literature [26,27].

The importance of training in obese subjects based upon the individual characteristics is well documented [13,25,28]. In fact, assessment of physiological capacity in obese subjects can allow to an individualized weight loss program based on individualized diet and physical activity. Cardiopulmonary exercise test (CPET), such as measurement of gas exchange during incremental test, remains the gold standard method to provide an accurate assessment of individual physiological responses induced by exercise. CPET is extremely important in clinical environment, to evaluate maximal and sub-maximal exercise intensities (e.g. individual ventilatory threshold and anaerobic threshold). Despite the confirmed validity of CPET, its application remains complex, expensive, and dependent on highly trained professionals. Moreover, CPET is not representative of functional capacity in real life. For instance, Houghton et al. [29] demonstrated that laboratory-based treadmill tests were not representative of heart failure patients’ capacities and their daily activities. Indeed, previous studies have used the 6-MWT for the purpose of evaluate obese subjects’ functional capacity [13,30]. The 6-MWT is an easy to administer test and un-expensive but it does not provide specific information when compared to CPET. 6-MWT may be used after training to assess the improvement of subjects’ fitness. For instance, Emerenziani et al. [13] used the 6-MWT every two months in order to check the subjects’ cardiorespiratory improvement. However, 6-MWT has some limitations when performed by special population such as obese and older subjects, due to their physical disability. In fact, severe obese condition may be associated with an imbalance body composition (fat mass > 45%), that may influence walking capacity during test.

However, 6-MWT has high correlation with workload, the intensity corresponding to maximal fat oxidation, and maximal oxygen uptake [30]. Makni et al. [30] published a regression model that allows the evaluator to estimate FAT_max_ from 6-MWT in obese children in both sexes. It might be very interesting in future studies to analyse the correlation between the HR at FAT_max_ and HR at IVT in obese adults. In fact, whether a correlation will be found, then the use of exercise intensity corresponding to IVT it might also be the best exercise intensity for fat oxidation.

In the present study, all subjects performed 6-MWT without any complications or premature conclusion of the test and the walking distance of both males and females was similar to data previously published [31]. As mentioned in the results section, males walked a longer distance than females, but the differences observed between sexes could be due to the difference in anthropometrics and body composition factors. In fact, males showed a lower %FM and higher height that may positively influence the 6-MWT results. To date, no equation allowing estimation HR_IVT_ from 6-MWD_HR_ across a large spectrum of obese subjects has been published. IVT allows to prescribe exercise intensity based on the real subject's capacity to perform physical exercise and it is useful to optimally prescribe correct exercise intensity in obese population [32,33,34]. Therefore, determination of HR_IVT_ from 6-MWT may be used for exercise intensity prescription in obese individuals, since it is easy to administer, un-expensive and can be routinely used.

Our regression model might be useful and suitable to all professionals that work in interdisciplinary teams to realize and optimize weight loss and weight management programs. By using stepwise multiple regression model analysis, we identified physiological variables that might predict HR at IVT. Our results demonstrate that HR during 6-MWT was the highest predictor of HR at IVT for both sexes whit a correlation coefficient equal to 0.79 and 0.78 for females and males respectively. The second predictor was HR at rest with a correlation coefficient equal to 0.60 and 0.56 for females and males, respectively. As expected, HR variable is strongly correlated with obesity and with fitness level; in fact, fat mass might decrease work capacity and increase HR at the same external workload. A lower HR_rest_ and 6-MWT_HR_ will result in a low HR_IVT_ that is an index of good fitness level. On the contrary, higher HR_rest_ and 6-MWT_HR_ will result in a high HR_IVT_ that is an index of low fitness level. The standard error of our predicted equation was 10.3 bpm and 10.4 bpm for females and males respectively. These results are in agreement with the study developed by Makni et al. [30] that found a predicted equation for maximal fat oxidation with a standard error equal to 18.5 and 10.2 for females and males respectively. In another study, developed by Arena et al. [35] the predicted equation for healthy subjects’ HR_max_ had a standard error of 11.4 bpm.

The use of an independent test set is considered the ‘gold standard' for assessing the predictive power of models, and is the most stringent and rigorous approach [23]. As previously described by Alexander et al. [23] the use of the coefficient of determination, R^2^, should be implemented with the root mean squared error or equivalent measures of dispersion in order to characterize the external predictively of a model.

The authors indicated that high R^2^, e.g. R^2^ > 0.6, ensures that the model fits the data well. In our equations, the R^2^ values were 0.69 for females and 0.65 for males. Moreover, RMSE from the cross-validation results should be low, approximately less than 10% of the range of target property value [23]. Our independent samples consist of sixty-six females and twenty-eight males. The corresponding RMSE was 7% in females, and 9% in males of the range of target property values. Therefore, we may affirm that our prediction equations perform well in females as in males.

Several authors [30,36,37] have previously studied the correlation between 6-MWT and other physiological parameters such as VO_2peak_ and FAT_max_. These authors, in their predictive models using 6-MWT, had included different physiological parameters (e.g., vital force capacity, systolic and diastolic pressure). We did not utilize these parameters, as we wanted to find a predicted equation from variables that can be easily assessed and can be used in subjects, as our obese individuals, who do not have cardiorespiratory impairments.

To the best of our knowledge, this is the first investigation carried out to predict HR at IVT from 6-MWT performance in obese adult of both gender. Although the CPET test remains the gold standard method for the measurement of physiological variables, this study suggests a valid alternative and an easier method to determine the HR corresponding to the exercise intensity at IVT, when CPET is unavailable or impractical. Nevertheless, when using this equation researchers and exercise professionals should be cautious to exclude obese subjects who have physical and health impairments potentially who might not correspond to the cohort that we have evaluated and characterized (age <65 years; %FM<45%).

### Study strong points

This is the first study aimed to establish a practical reference equation to estimate the heart rate at individual ventilatory threshold in obese adults.

HR at rest and the HR during 6-MWT might be used to well predict HR corresponding to IVT in both genders.

This practical reference equation is a valid alternative method to determine the HR corresponding to the optimal exercise intensity for healthy purposes.

### Study limitations

Even if the method to assess the IVT by plotting the ventilatory equivalent of oxygen (VE/VO_2_) as a function of VO_2_ is scientific validated, different approaches including other variables and visual inspection may be used for future studies.

We are aware that during 6-MWT we did not assess all physiological parameters (e.g. blood pressure). Therefore, future studies may implement the current equation with new parameters in order to reach the highest correlation. Moreover, due to the specific studied population we used 50m corridor in order to decrease the number of changes of direction performed during the 6MWT by the subjects. This choice was taken in order to avoid muscle and ankle problems in subjects affected by obesity. In conclusion, the current study findings demonstrate that in obese adults, the HR at rest and the HR during 6-MWT might be used to well predict HR corresponding to IVT.

## Supporting information

S1 File Dataset. HR_rest_ = heart rate at rest, 6-MWTHR = average heart rate during six minute walking test; HR_IVT_ = heart rate at IVT; Gender: 0 = females, 1 = males.
(XLSX)
